# Neuromuscular Adjustments Following Sprint Training with Ischemic Preconditioning in Endurance Athletes: Preliminary Data

**DOI:** 10.3390/sports9090124

**Published:** 2021-09-02

**Authors:** Stéphan Bouffard, Pénélope Paradis-Deschênes, François Billaut

**Affiliations:** 1 Department of Kinesiology, Laval University, Quebec, QC G1V 0A6, Canada; stephan.bouffard.1@ulaval.ca (S.B.); penelope.paradis-deschenes.1@ulaval.ca (P.P.-D.); 2 Institut Universitaire de Cardiologie et de Pneumologie de Québec, Québec, QC G1V 4G5, Canada

**Keywords:** blood-flow restriction, HIIT, hypoxia, NIRS, peripheral adaptation

## Abstract

This preliminary study examined the effect of chronic ischemic preconditioning (IPC) on neuromuscular responses to high-intensity exercise. In a parallel-group design, twelve endurance-trained males (VO_2_max 60.0 ± 9.1 mL·kg^−1^·min^−1^) performed a 30-s Wingate test before, during, and after 4 weeks of sprint-interval training. Training consisted of bi-weekly sessions of 4 to 7 supra-maximal all-out 30-s cycling bouts with 4.5 min of recovery, preceded by either IPC (3 × 5-min of compression at 220 mmHg/5-min reperfusion, IPC, *n* = 6) or placebo compressions (20 mmHg, PLA, *n* = 6). Mechanical indices and the root mean square and mean power frequency of the electromyographic signal from three lower-limb muscles were continuously measured during the Wingate tests. Data were averaged over six 5-s intervals and analyzed with Cohen’s effect sizes. Changes in peak power output were not different between groups. However, from mid- to post-training, IPC improved power output more than PLA in the 20 to 25-s interval (7.6 ± 10.0%, ES 0.51) and the 25 to 30-s interval (8.8 ± 11.2%, ES 0.58), as well as the fatigue index (10.0 ± 2.3%, ES 0.46). Concomitantly to this performance difference, IPC attenuated the decline in frequency spectrum throughout the Wingate (mean difference: 14.8%, ES range: 0.88–1.80). There was no difference in root mean square amplitude between groups. These preliminary results suggest that using IPC before sprint training may enhance performance during a 30-s Wingate test, and such gains occurred in the last 2 weeks of the intervention. This improvement may be due, in part, to neuromuscular adjustments induced by the chronic use of IPC.

## 1. Introduction

As athletes advance in competitive level, improvements in performance become smaller and harder to achieve. In the continuous search to optimize training adaptations and performance, ischemic preconditioning (IPC) has attracted recent interest in enhancing otherwise-reliable training methods. While IPC has been shown on multiple occasions to enhance physiological responses and both aerobic and anaerobic performances acutely [1], the ergogenicity of its chronic use remains to be examined consistently before making practical training recommendations. Resting application of IPC for 3 to 7 days can improve VO_2_max, maximal aerobic power (MAP) and performance during repeated Wingate tests [2] and repeated swimming sprints [3] in an active population. Such improvements are mostly derived from blood flow and O_2_ kinetics changes [4,5,6,7,8]. However, this is not always the case, especially in an athletic population. For instance, 3 to 9 days of IPC failed to enhance 4 and 5-km time-trial performances in trained individuals [9,10] and 600-m on-ice time trial in elite speed skaters [11].

In athletes, acute and chronic applications of IPC at rest may be unlikely to elicit significant performance adaptations since this population has already maximized several key physiological functions [12]. However, since IPC can increase exercise intensity, it may very well enhance training load during training, which, when applied repeatedly, could enhance physiological adaptations. Some authors have therefore attempted to perform compressions immediately before exercise to manipulate vascular and metabolic stress. IPC used prior to high-intensity training sessions (tempo runs) for 8 weeks failed to enhance VO_2_max and 1-km time-trial running performance in distance runners compared to the same training with placebo compressions [9]. On the other hand, our laboratory reported ergogenic effects of 4 weeks of IPC applied before sprint-interval training (SIT) sessions on 5-km cycling time-trial performance in endurance athletes [13]. The discrepancy in these conclusions may be due to the different IPC procedures (unilateral vs. bilateral), probably inducing a different vascular/metabolic cascade and the intensity of the training sessions (tempo vs. “all-out” efforts). In fact, high power output at the onset of exercise and repeated metabolic stress might be the two most important aspects when it comes to creating adaptations with SIT [14,15], and since IPC can improve key qualities of the 30-s Wingate test such as maximal concentric force [6,16] and peak and mean power output [2,3], its chronic use along this particular sprint training modality appears to be a promising way of improving performance in athletes.

The enhancement in high-intensity exercise performance may originate from various sites along the pathway from the central nervous command to intramuscular contractile machinery sites [17,18]. If vascular, metabolic, and contractile responses to IPC have been explored, the neuromuscular activation of a contracting muscle, which is a hallmark characteristic from sprint exercise, is still poorly understood. Information about muscle electrical activity (EMG), both in amplitude (root mean square) and frequency (median power frequency), would provide further knowledge about the effects of IPC on muscle recruitment and the development of neuromuscular fatigue. Data from an animal model showing higher EMG amplitude after IPC (60-min of compression at 300 mmHg followed by 120-min of recovery) concomitant with a simultaneous increase in maximal isometric strength in feline hind limbs suggest an enhanced motor-unit recruitment [19]. Although little data are currently available in humans, research suggests that IPC can alter muscular recruitment patterns in trained muscles. Acute IPC was shown to increase EMG amplitude and spectrum frequency during short-duration bicycle sprints [20,21], and investigations of resistance exercise performance have quite consistently reported strength enhancement following IPC [16,22], but without measuring muscle electrical activity.

Therefore, the goal of this study was to examine the effects of the combination of IPC and SIT on power output, fatigue, and neuromuscular activity during maximal sprint cycling in endurance-trained athletes. Based on the above literature, we hypothesized that sprint training preceded by IPC would increase muscle recruitment and the preferential selection of higher-threshold motor units (assessed via the EMG signal), and that such neuromuscular adjustments would increase peak and mean power output and attenuate the fatigue index more than training alone.

## 2. Materials and Methods

### 2.1. Participants

This investigation was part of a larger project examining the impact of IPC on endurance performance adaptations and blood markers of angiogenesis and hypoxic signaling. These data have been published elsewhere [13]. Since short-term endurance performance can also be influenced by anaerobic capacity, we also investigated the effects of training with IPC on this endurance performance determinant and the concurrent neuromuscular function changes. The current research question was still determined a priori and prospectively studied as a separate question.

Fifteen participants completed the study, but complete sets of mechanical and neuromuscular data on all investigated muscles were kept on twelve of them due to methodological issues with EMG data acquisition and analysis. Twelve participants were determined sufficient by power analysis with JMP software (version 10, Cary, NC, USA) to detect a difference in primary outcome variables over time between conditions, with an alpha level of 0.05 and a desired power of 0.90 and based on the average effect (d = 0.22) observed in our laboratory with similar equipment [6,11,13]. Participants were healthy endurance-trained male cyclists, triathletes, and runners with experience in cycling (6.5 ± 0.5 h/week of training at the time of study, VO_2_peak 60.0 ± 9.1 mL·kg^−1^·min^−1^, age 29.6 ± 9.9 years, mass 75.38 ± 9.71 kg), with at least 2 years of training experience in their respective sport. They competed at the regional and national levels and were in their pre-season phase. None of the participants use any tobacco/nicotine products or take any medication. The study was approved by the Ethics Committee of University Laval (certificate #2016-093), while also respecting the principles found in the Declaration of Helsinki. Informed consent, in written form, was provided by the participants after being informed of the potential risks and benefits associated with the research protocol and its interventions.

### 2.2. Experimental Design

Participants visited the laboratory a total of 13 times, including eight biweekly training sessions, spread over the course of 4 weeks. The other five visits were used for pre- (3), mid- (1), and post-training (1) evaluations. Participants typically trained in the morning (7–9 a.m.) or in the late afternoon (4–6 p.m.), but training and testing times were the same during the intervention for every participant to avoid the influence of circadian rhythms. Prior to each testing and training day, vigorous exercise was avoided for 48 h and alcohol and caffeine were refrained from for 24 h. Sessions were separated by at least 48 h. Temperature (21.5 ± 0.1 °C) and humidity (29.9 ± 1.0%) were kept stable across training and testing sessions. Participants were assigned to either IPC or PLA using a between-groups design based on age, VO_2_peak, and peak power output (PPO) from the Wingate test. Training intervention was the same for both the IPC and PLA groups (see training intervention). Retesting of the Wingate test occurred 2 to 4 days after the fourth SIT session (mid-), and after the end of training (post-).

### 2.3. Training Intervention

The 4-week training program consisted of two SIT sessions per week, in which all-out sprints of 30 s separated by 4.5 min of rest (4-min passive; 0.5-min active) were performed with no pacing. Training volume followed a weekly progressive increase with four repetitions per workout in week 1, five in week 2, six in week 3, and finally seven in week 4. Every training session took place at the high-performance training center of Université Laval, using Keiser M3+ cycle ergometers (Keiser corporation, Fresno, CA, USA), and was preceded by either the IPC or the PLA protocol (see Section 2.4 for procedure details). All sessions were supervised by an investigator. Participants used the same ergometer with the same parameters for every workout (resistance, handlebars, and seat settings). Every training session followed the same protocol: IPC or PLA treatment, 10-min standardized warm-up, 2-min seated rest, 30-s repeated maximal cycling training, and 5-min cool-down. The sprints were all initiated as described in the testing session (Section 2.5) with an additional 15-s of cycling without resistance thereafter and a 4-min passive rest before the next repetition.

To monitor internal training loads of sessions, peak and minimum power, as well as RPE, were recorded by the investigator during all sprints. Session RPE was then calculated (RPE score × duration) to compare training load between groups [23]. Participants also kept a training log to track the training load (RPE and duration) of their usual non-prescribed training regimen, and were asked to reproduce these sessions for the duration of the study. Participants were also asked to replace their usual high-intensity sessions with the prescribed SIT sessions.

### 2.4. Ischemic Preconditioning

In order to minimize the placebo effect, participants were told that the aim of the study was to compare two different cuff pressures that could impact training and performance positively, with varying effects on blood flow related to each pressure applied. The researchers rapidly inflated a non-elastic nylon blood pressure cuff (WelchAllyn, Skaneateles Falls, NY, USA, width: 21 cm) installed on each upper thigh. Pressure of either 220 mmHg for IPC or 20 mmHg for PLA was applied for 5 min on one leg, then released when the other leg was being compressed for 5 min. The process was repeated 3 times, allowing 5-min of reperfusion between compressions to each leg, adding up to a total IPC time of 30 min [13]. Participants in the IPC group were familiarized with the procedure during the familiarization session. The IPC and PLA procedures were performed within minutes before every training session in order to replicate the reality of training where athletes prime and warm themselves up before exercise. 

### 2.5. Testing Procedures and Data Collection

#### 2.5.1. Baseline

Resting heart rate (HR) and blood pressure were recorded while participants were sitting on a couch (inclusion criteria <100 bpm and <140/90 mmHg). Anthropometric data were collected under the form of height, body mass, and thigh skinfold thickness (IPC: 5.3 ± 0.7 mm; PLA: 6.7 ± 0.6 mm) and circumference (IPC: 55.6 ± 1.2 cm; PLA: 54.9 ± 1.2 cm). Body fat percentage was measured (Tanita TBF-310; Tanita Corp. of America Inc., Arlington 157 Heights, IL, USA). At that session, peak O_2_ consumption was also measured. While seated on an electromagnetically braked cycle ergometer (Excalibur Sport, Lode, The Netherlands), participants remained in position for a 2-min baseline, then executed a 5-min warm-up at 100 W, prior to finally performing a maximal step test (30 W increase per minute, up to volitional fatigue). In order to evaluate VO_2_, carbon dioxide production (VCO_2_), and respiratory exchange ratio, we analyzed exhaled gases during the whole test (Breezesuite, MedGraphics Corp., Saint Paul, MN, USA). The greatest average collected for a 20-s segment was identified as VO_2_peak. A decremental 5-min cool-down concluded the testing session, with the participants cycling at 100 W and gradually decreasing intensity by 25 W per minute. 

#### 2.5.2. Familiarization

The familiarization session for the Wingate test was nearly the same as the actual pre-testing described in Section 2.5.3, with the exception that no analysis devices were used and no data were collected. Every participant also personalized their handlebar and seat settings on all cycling apparatus for a seated position (Velotron and Keiser ergometers). Since changes in seat tube angle, crank length, and saddle height can affect physiological and biomechanical parameters, all parameters were kept constant for all training and testing sessions (stationary bike model, resistance level, settings for handlebars and saddle) [24].

#### 2.5.3. 30-s Wingate Test

The pre-, mid-, and post-training testing sessions were identical, and consisted of a 10-min warm-up and completion of one Wingate test on a computer-controlled electrically braked cycle ergometer (Velotron Elite, RacerMate, Seattle, WA, USA) equipped with toe-clips. Participants warmed-up for six minutes at a self-selected cadence with a low resistance adjusted with the gear ratio on the ergometer, followed by three 5-s accelerations separated by 15 s active recovery with increasing gear ratios to elicit 85, 95, and 100% of subjective maximal effort. After these accelerations, participants completed the warm-up at their chosen pace and resistance. These gear ratios and corresponding power outputs were continuously noted by the experimenter and strictly reproduced thereafter. After two minutes of rest, the Wingate test was carried out. A 20-s gradual increase in cadence was used to attain 100 W, then participants had three seconds to accelerate and reach peak power in the shortest possible time. Using a computer (Wingate Software Version 1.11, Lode BV, Groningen, The Netherlands), a resistance corresponding to 7.5% of the participant’s bodyweight was applied throughout the 30-s maximal effort. Participants remained seated at all times. Food intake and physical activity were logged into a journal by the participants at every test. Then, they were asked to replicate their eating and exercise habits for 24 h and 72 h before testing, respectively.

Power output data were sampled at 10 Hz throughout the test on a Velotron cycle ergometer. The greatest power value recorded over a 1-s interval was identified as PPO. Mean power output (MPO) was identified as the average output over the course of the 30 s of the test. Power output data were also segmented in six 5-s time intervals (0:5, 5:10, 10:15, 15:20, 20:25, and 25:30 s). For each segment, MPO was calculated and expressed as a percentage of the first segment’s value. Using these percentages, a fatigue index was calculated as:

FI=([greatest percentage−smallest percentage]/greatest percentage) × 100

To determine subjective perceived exertion, the Borg 10-point scale was used. RPE was collected after every Wingate test [25].

#### 2.5.4. Electromyography Recording and Analysis

During every Wingate test, the EMG signals of the biceps femoris, vastus lateralis, and gastrocnemius muscles were recorded from the dominant leg with surface electrodes (Delsys, Trigno Wireless, Boston, MA, USA). The EMG signal was pre-amplified, filtered (bandwidth 12–500 Hz, gain = 1000, sampling frequency 2 kHz) and recorded with EMGWorks Acquisition Software (Bagnoli EMG System; Delsys, Inc., Natick, MA, USA). It was then exported to a personal computer for subsequent filtering and analysis on Analyse Software (Université Laval, Québec, QC, Canada), which uses Matlab routines. The three electrodes were placed longitudinally on the muscle bellies in accordance with recommendations from SENIAM [26]. Prior to electrode installation, skin was shaved, lightly sanded, then cleaned with an alcohol swab. To assure constant placement of the electrodes, all muscle sites were marked with a water-proof permanent marker and maintained during the study.

The EMG recording was manually initiated on the computer immediately before the 23-s acceleration phase that preceded the Wingate test, and stopped after the test was over. To match power output data analysis, the 30-s EMG signals were segmented into six 5-s time intervals. The root mean square of each of the three muscle signals was calculated for each time interval. The frequency spectrum of each time interval was analyzed using a fast Fourier transformation. The frequency spectrum was restricted to frequencies in the range 5–500 Hz, as the EMG signal content outside of this range consists mostly of noise. The median frequency was then determined. Then, the root mean square and median frequency obtained for every muscle were averaged together for every time interval to obtain one parameter per interval representing a global index of activation [27]. Finally, both root mean square and median frequency values were normalized to the value of the first time interval for every test.

### 2.6. Statistical Analysis

We evaluated the magnitudes of difference within groups from pre- and mid-training to post-training for all physiological and performance variables, and the percentage difference between physiological and performance changes in IPC and PLA throughout the six 5-s segments of the Wingate test and the entire test. We assessed the practical significance of using IPC compared with PLA with Cohen’s effect sizes (ES) ± 90% confidence limits and comparisons to smallest worthwhile changes that were calculated as the standardized mean differences of 0.2 between-subject standard deviations [28,29] for all variables. Prior to analysis, all data were log-transformed. Mechanistic inferences were employed to evaluate the effects of all variables. Benefits were labeled as likely (75 to 95%), very likely (95 to 99.5%), or almost certain (>99.5%). However, whenever both the possibilities of positive and negative effects of IPC surpassed 5%, changes were described as unclear [28,29]. Data are presented as mean ± standard deviation (SD).

## 3. Results

All participants completed the eight training sessions and tolerated the IPC without complication. 

### 3.1. Mechanical Data

Peak and mean power output and fatigue index are displayed in Table 1. The changes in PPO after two and four weeks of training appear slightly greater in PLA (↑7.4 ± 11.8%, ES 0.48, chances to observe greater/trivial/lower score 85/13/1%) than IPC (↑3.9 ± 11.2%, ES 0.16, 37/62/1), but there was no clear difference between groups. Similarly, MPO did not clearly change between groups (IPC: ↑1.3 ± 5.5%, ES 0.08, 13/86/1% vs. PLA: ↑1.9 ± 1.6%, ES 0.12, 1/99/0%, group difference: −0.6 ± 3.1%, ES −0.04, 3/90/8%).

**Figure 1:**
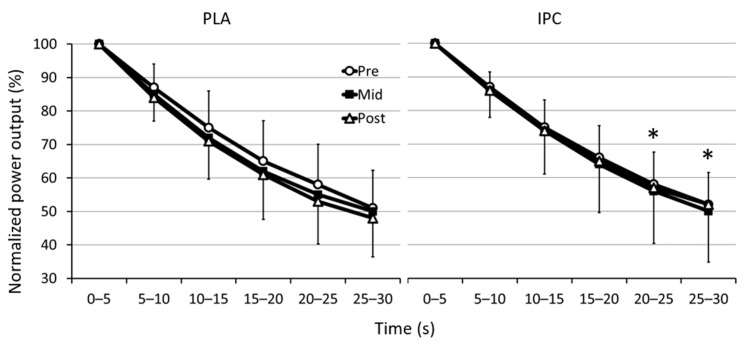
Changes in mean power output (MPO) during the six 5-s segments of the Wingate test with placebo (PLA) and ischemic preconditioning (IPC) before (pre), after 2 weeks of training (mid), and after 4 weeks of training (post). * Clear difference between groups (see text for details).

The mean power output developed during the six 5-s intervals of the Wingate test before and after training in both groups is displayed in Figure 1. From mid- to post-, mean power output was likely enhanced in IPC for interval 20:25 s (IPC: ↑1.4 ± 6.7% vs. PLA: ↓5.8 ± 15.7%, group difference: 7.61 ± 10.0%, ES 0.51, 79/17/4%) and for interval 25:30 s (IPC: ↑2.9 ± 7.1% vs. PLA: ↓5.4 ± 17.9%, group difference: 8.76 ± 11.2%, ES 0.58, 81/14/4%). 

From pre- to post-, no clear difference was noted in the fatigue index between groups (Table 1). However, from mid- to post-, it decreased in IPC (↓5.8 ± 10.0%), while it increased in PLA (↑4.6 ± 15.1%), yielding a clear group difference in favor of training with IPC (−10.0 ± 10.2%, ES −0.46, 1/14/85%).

### 3.2. Electromyographic Activity

**Figure 2:**
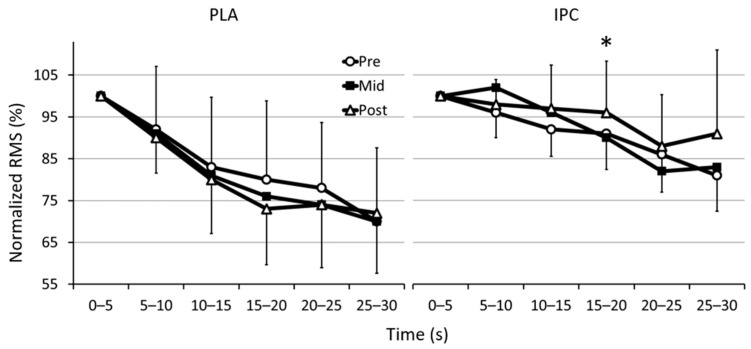
Changes in normalized EMG amplitude (root mean square, RMS) of the biceps femoris, gastrocnemius, and vastus lateralis muscles during the six 5-s segments of the Wingate test with placebo (PLA) and ischemic preconditioning (IPC) before (pre), after 2 weeks of training (mid), and after 4 weeks of training (post). * Clear difference between groups (see text for details).

Figure 2 displays normalized root mean square for the six 5-s intervals of the Wingate tests before and after training. Overall, the root mean square declined on average by 20–30% over the course of the Wingate with developing neuromuscular fatigue. However, from pre- to post-, there was a clear change in root mean square during interval 15:20 s (IPC: ↑5.2 ± 12.9% vs. PLA: ↓8.3 ± 17.9%, group difference: 14.7 ± 16.5%, ES 0.80, 88/9/4%), with all muscle groups showing similar contributions to this change in recruitment. In the remaining intervals, the changes between groups remained non-significant. 

**Figure 3:**
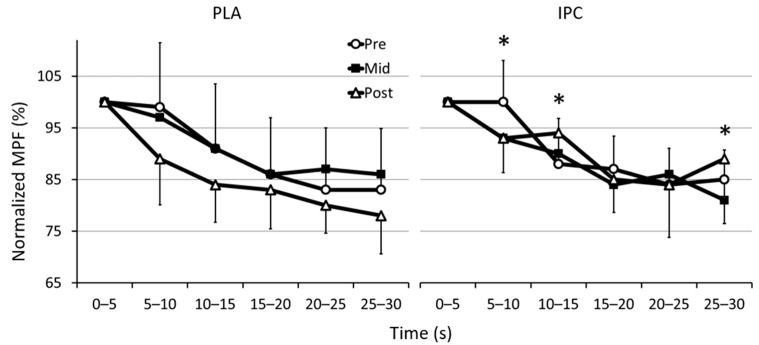
Changes in normalized median power frequency (MPF) of the biceps femoris, gastrocnemius, and vastus lateralis muscles during the six 5-s segments of the Wingate test with placebo (PLA) and ischemic preconditioning (IPC) before (pre), after 2 weeks of training (mid), and after 4 weeks of training (post). * Clear difference between groups (see text for details).

Figure 3 displays the normalized EMG mean frequency during the Wingate tests before and after training. Spectral indices were compressed by 15–20% over the course of the Wingate with developing neuromuscular fatigue. However, compared with root mean square scores, between-group differences were observed in frequency behavior from pre- to post-training and from mid- to post-training. From pre- to post-, median frequency clearly increased in IPC in interval 10:15 s (IPC: ↑7.0 ± 12.6% vs. PLA: ↓7.8 ± 11.5%, 16.0 ± 12.8%, ES 1.42, 96/3/2%). Similar changes in frequency were also observed from mid- to post- in interval 5:10 s (IPC: ↑0.7 ± 12.7% vs. PLA: ↓8.1 ± 8.4%, 9.6 ± 11.6%, ES 0.88, 87/9/5%), interval 10:15 s (IPC: ↑4.6 ± 15.7% vs. PLA: ↓8.4 ± 9.0%, 14.2 ± 13.8%, ES 1.27, 93/4/3%), and interval 25:30 s (IPC: ↑9.9 ± 12.8% vs. PLA: ↓8.9 ± 10.3%, 20.6 ± 12.3%, ES 1.80, 99/1/0%). The gastrocnemius and biceps femoris muscles were the main individual contributors to these changes in neuromuscular activity in the lower body as a whole, with no difference in frequency observed in the vastus lateralis.

## 4. Discussion

This study examined the potential of IPC to enhance performance gains during supra-maximal exercise following a SIT regime in trained individuals, as well as the underlying neural mechanisms responsible for these gains. The main finding was that in endurance athletes, when compared to training alone, 8 sessions of SIT preceded by 3 cycles of bilateral occlusions improved fatigue resistance during a Wingate test, as observed from greater power output in the last 10 s of the test and the lower fatigue index. These benefits occurred in the second half of the training. Although the adaptive responses to sprint performance are complex, the current preliminary study presents some evidence to suggest that the recruitment of high-frequency motor units was better maintained with IPC. 

Anaerobic capacity is one of the key components of endurance performance, and athletes develop that quality with high-intensity interval training [30]. Among the varied training programs, SIT has been used quite extensively and leads to significant benefits in maximal power and endurance [14,15,30]. Acute IPC can increase performance during a supra-maximal exercise, and one may therefore argue that this maneuver could further enhance physiological adaptations and performance when applied chronically during training. While this combination has proven successful to enhance endurance time-trial performance [13], it remains poorly understood whether it can also enhance anaerobic capacity and sprint performance. Acute studies have reported improvement of power output during short (6 s [20]), medium (15–30 s [2,3]) and long (60 s [21]) sprints, as well as improvement of key qualities associated with sprinting such as maximal muscle concentric force [6,16]. That being said, IPC has also been reported to fail to increase power output during sprints [31,32,33]. Methodological considerations across studies such as the IPC protocol used, the absence of individualization of the occlusion pressure, the IPC-to-exercise timing, and the individual response to hypoxic/vascular stress may explain the above differences, which have been proposed elsewhere [34].

The hypoxic/vascular stress induced by an acute IPC application might also not be sufficient to alter performance in athletes who have spent several years developing all the systems involved in energy production. When applied chronically and immediately before exercise, IPC may enhance the physiological stress and lead to greater performance changes over several training sessions. In the present study, 3 cycles of IPC at 220 mmHg performed before biweekly SIT sessions led to greater mean power output in the final stages of a Wingate test. These findings suggest that IPC attenuated the development of fatigue during the sprint (~10% difference in the fatigue index). While these are the first data to suggest beneficial changes to supra-maximal anaerobic exercise performance, IPC has already been shown to enhance performance during high-intensity resistance exercise. Carvalho and colleagues [22] demonstrated a greater number of knee extensions performed at 75% of 1 RM after 6 weeks of training combined with IPC in resistance-trained participants. With only a handful of training studies currently available, future research will have to ascertain these findings to refine practical training recommendations. In addition, although the current IPC maneuver was performed before training, we cannot exclude the influence of the second window of protection that typically develops over several hours/days after the conditioning stimulus [35]. This delayed phase of conditioning is mediated by different inflammatory and gene expression mechanisms and cannot be distinguished from the initial time window in the current study. Further studies will have to disentangle this aspect especially in the athletic field where athletes use ergogenic strategies over a relatively long period. Taken together, these data suggest that IPC may be incorporated to high-intensity training in varied athletic populations to increase performance outcomes. 

Neuromuscular fatigue develops rapidly during supra-maximal exercise due to the large contribution from the glycolytic metabolism to ATP phosphorylation and ensuing accumulation of metabolic by-products [36,37]. The decline in power output measured during the Wingate test was similar to that reported in the literature, totaling ~50% [38,39]. Determinants of Wingate performance include the muscle buffer capacity, the regulation of pH, the production of energy from anaerobic pathways, and muscle recruitment patterns [36,39]. According to our hypothesis, the improved power output in the latter stages of the Wingate test in the IPC training group coincided with neuromuscular adjustments, namely a lower attenuation of the EMG signal median frequency. Root mean square and frequency reflect the neuromuscular activity (motor unit recruitment and firing rate) and the type of motor units recruited, respectively [17]. Acute IPC has been shown to increase EMG amplitude and spectrum frequency during short-duration bicycle sprints [20,21], but its effects on chronic adaptations of the neuromuscular system are limited. In the present study, the lack of impact on EMG amplitude is unclear but may be related to the maximal intensity of the Wingate test, making it difficult to recruit additional muscle fibers in well-trained and motivated athletes. Alternatively, since IPC was performed during training only and not during application of the Wingate test, one may also argue that its ergogenic effects on muscle recruitment act relatively rapidly (i.e., within minutes to hours after the maneuver) and that they dissipate over time. In fact, Wingate testing occurred a few days after IPC in the present study, suggesting that the neuromuscular impact of a chronic use is limited. Nonetheless, our data are in agreement with recent findings (albeit still acute) of a lack of change in voluntary activation and EMG amplitude after sustained maximal isometric contractions [40] or in spinal reflex pathways after repeated short cycle sprints [41]. Further research using electrical or magnetic stimulation to quantity adaptations of the nervous system after sprint training is necessary.

High rate of muscle fatigue and spectral compression are typical signs of an all-out Wingate sprint [36,39]. This spectral shift indicates a preferential recruitment of more endurant, smaller motor units due to early fatigue in their type-II counterparts [42,43], and results in a decrease in power [44]. The decrease in stimulation frequency is caused by the accumulation of metabolites (H^+^ and K^+^ ions, inorganic phosphates) and the ensuing fall in pH, which stimulate type III/IV afferents that reflexively signal the CNS to selectively recruit less-fatigable skeletal muscle fibers. Interestingly, the autacoids (e.g., adenosine, bradykinin, opioids) released during repeated IPC cycles are thought to attenuate the stimulation and signaling of type III/IV afferent groups, which causes a disruption to the central feedback loop mechanism [18,45,46,47,48,49]. This phenomenon could explain how IPC preserved the median frequency towards higher frequencies and limited the drop in mean power output post-training. While these adjustments occurred after 2 weeks, the main changes manifested with 2 more weeks of training combined with IPC. The reason why such changes primarily occurred within the gastrocnemius and biceps femoris, two bi-articular muscles, remains unclear.

Some limitations should be noted in this preliminary investigation, which should be addressed in future research on this topic. First, the training and testing of anaerobic power were performed on different ergometers to facilitate the study logistics and allow group training with multiple ergometers. However, there is the possibility that participants were less accustomed to sprinting on the Velotron ergometer during and after the training period, which could have influenced the ability to quickly generate high power outputs and, therefore, detect the subtle changes induced by IPC. Nonetheless, this issue was the same for both groups, but should be addressed in other studies. Second, the sample size was relatively small (*n* = 12), and statistical power could have been lacking to detect subtle training-induced changes, especially after only 8 training sessions. Even though the chosen statistical approach is well-suited for the analysis of low sample size data [29] and we were able to detect clear differences between training groups in the second half of the training, conclusions must still be taken with caution. Finally, the current data are also limited to the use of surface EMG, and future research will need to robustly examine training-induced nervous system adaptations with stimulation techniques. 

## 5. Conclusions

We concluded from these preliminary findings that endurance athletes exhibited higher fatigue resistance during a Wingate test after four weeks of SIT training with application of bilateral IPC. This improvement may be due to neuromuscular adjustments induced by the chronic use of IPC, especially a maintenance of high-frequency motor units of the targeted muscles. This strengthens the rationale for using IPC to augment training stimulus during a high-intensity training regimen. Coaches and sport scientists looking to improve the anaerobic component of endurance performance may use IPC before sprint training.

## Figures and Tables

**Table 1:** Mean changes in performance in the Wingate test preceded by ischemic preconditioning (IPC) or placebo (PLA) before (pre), after 2 weeks of training (mid), and after 4 weeks of training (post).

	IPC			PLA			IPC vs. PLA		
	PRE	MID	POST	PRE	MID	POST	PRE-MID	MID-POST	PRE-POST
	Mean ± SD			Mean ± SD			%D (ES)		
**PPO** **(W)**	1077.6 ± 208.0	1122.0 ± 251.2	1131.4 ± 273.8	1008.3 ± 84.9	1052.9 ± 139.4	1093.6 ± 185.2	−0.4% (−0.03)	−2.9% (−0.18)	−3.3% (−0.21)
**PPO/kg** **(W/kg)**	14.1 ± 2.1	14.4 ± 2.1	14.9 ± 2.9	13.7 ± 1.8	14.1 ± 1.8	14.9 ± 2.0	−0.5% (−0.26)	−1.3% (−0.24)	−3.5% (−0.24)
**MPO** **(W)**	750.5 ± 115.4	751.0 ± 114.1	759.1 ± 105.5	704.9 ± 101.1	708.5 ± 108.4	717.6 ± 97.1	−0.3% (−0.02)	−0.2% (−0.02)	−0.6% (−0.04)
**MPO/kg** **(W/kg)**	9.9 ± 1.3	9.9 ± 1.2	10.0 ± 1.1	9.5 ± 0.9	9.5 ± 1.2	9.7 ± 0.9	−0.2% (−0.03)	−0.5% (−0.05)	−0.7% (−0.06)
**Fatigue index (%)**	48.1 ± 9.6	50.4 ± 15.0	48.4 ± 17.1	48.8 ± 11.3	49.9 ± 10.7	52.3 ± 11.6	−0.6% (−0.02)	**−10.0% (−0.46)**	−10.5% (−0.49)

Note: Abbreviations: CL, confidence limits; %D, percentage difference between changes in IPC and PLA; ES, effect size; MPO, mean power output; NA, not available; PPO, peak power output; SD, standard deviation. Clear differences between IPC and PLA are indicated in bold.

## Data Availability

The supporting dataset is available upon request to the corresponding author.
